# New drugs are not enough: addressing social determinants as a critical component of drug-resistant TB care

**DOI:** 10.5588/ijtldopen.25.0075

**Published:** 2025-04-09

**Authors:** D. Giovenco, N.S. Shah, K. Ansorge, D. Operario, N.R. Gandhi

**Affiliations:** ^1^ Emory University, Rollins School of Public Health, Atlanta, GA, USA;; ^2^ Emory University, School of Medicine, Atlanta, GA, USA.

**Keywords:** DR-TB, social determinants of health, person-centered care, social epidemiology

## Abstract

The global fight against drug-resistant TB (DR-TB) is hindered by an underappreciation of the importance of the social determinants of TB. Although shorter, all-oral regimens have improved treatment options, high rates of non-adherence persist due to stigma, financial hardship and other healthcare access barriers. Lessons from HIV highlight the importance of addressing structural and psychosocial factors in treatment management. Consideration of an ecosocial framework integrating biological, social and environmental perspectives is critical for effective DR-TB interventions. A paradigm shift toward patient-centered care and social support strategies is urgently needed to improve treatment outcomes and advance global efforts to eradicate DR-TB.

The emergence of drug-resistant TB (DR-TB) is a significant challenge to global public health, with nearly half a million new cases each year.^1,2^ Despite advances in diagnostics and treatment, the fight against DR-TB remains stymied by the protracted process of drug development and scale-up, which has seen only a handful of new therapeutics reach patients in the past decade. The introduction of bedaquiline (BDQ) and approval of the BPaL(M) regimen marked a pioneering moment. However, the shift from 18+ month, injectable-containing regimens to all-oral, 6-month, or 9-month regimens has not been sufficient to curb the DR-TB epidemic.^3^ Within a decade of BDQ’s availability, resistance has already surfaced,^4^ and rates of treatment interruption remain high (10% in a 2021 cohort), despite simpler, shorter and less toxic DR-TB treatment regimens.^2^ We propose that interventions must urgently address the social determinants of DR-TB care^5,6^ and learn from other disease areas. For example, over the past two decades, interventions for HIV were designed specifically to mitigate the social and structural barriers faced by marginalized populations and have proven effective in enhancing treatment adherence.^7,8^ This prompts us to critically reflect on our prevailing strategies in tackling DR-TB and ask, ‘are we focusing our resources and efforts in the right places?’

## The complexities of DR-TB treatment

The range of challenges that can lead to DR-TB treatment interruption is significantly underappreciated.^9,10^ Starting from diagnosis through treatment initiation and discharge, patients face a myriad of challenges that can affect treatment continuity. The treatment cascade can often be non-linear, characterized by recurrent lapses and re-engagements in care,^11^ described as ‘churn’, a concept frequently referenced in the context of HIV treatment.^12^ This churn emphasizes the importance of sustained retention in care as a determinant of favorable clinical outcomes. It underscores the need for tailored interventions that accommodate the intricacies of individual experiences and social and environmental contexts throughout the length of treatment,^13^ a core pillar of the WHO End TB Strategy. Historically, efforts to combat DR-TB have centered on improving drug development, optimizing treatment protocols and supporting adherence. Although these are undeniably vital aspects, they often overlook the overarching social and structural factors that contribute to treatment interruption and non-adherence. For example, social factors, including stigma, social support, employment status, and community-level information and norms surrounding TB, may shape an individual's ability to navigate the healthcare system and complete long and complex TB treatment regimens. Structural factors, which encompass the broader systemic and institutional conditions that create barriers to equitable care, including healthcare accessibility, availability of treatment services, policy environments, food security, and housing availability, may determine whether a person is diagnosed with TB early, can afford transportation to a clinic and receives sustained treatment support. To effectively counteract DR-TB, we must shift our focus toward these interconnected, person-centered influences, creating a more effective and equitable DR-TB response that transcends solely biomedical solutions. One such trial is underway in South Africa that will provide integrated psychosocial and adherence support informed by individual needs with the goal of maximizing treatment success of new DR-TB regimens.^14^

## A sociobehavioral lens on treatment adherence

Social epidemiology seeks to identify the factors that increase a population’s risk, or enhance the vulnerability of contracting a disease, with a focus on policy and program interventions that address population-level fundamental social determinants of disease.^15,16^ The barriers to treatment adherence are not solely medical, but are deeply rooted in the social determinants of health (SDoH). In the context of DR-TB, the interplay between socioeconomic factors, healthcare access, and the environmental context significantly influences a patient’s journey from diagnosis to successful completion of TB treatment.^9^ Although the global TB community has recognized the importance of addressing social determinants for over a decade, progress has remained insufficient, particularly in the development of effective interventions.^17^ In 2024, WHO guidance on social protection for people affected by TB provided an important framework for countries to move from the political commitment made during the 2018 and 2023 United Nations (UN) High-Level Meetings on TB to meaningful action.^18^ Early evidence supports patient-centered care as an effective approach for substantially reducing loss to follow-up, as demonstrated in a cohort of people with multidrug-resistant TB receiving short, all-oral regimens in Afghanistan.^19^ Examples of patient-centered care in DR-TB management include adherence counseling, psychosocial and economic support, transportation assistance, and decentralized or community-based care models that reduce the need for frequent clinic visits. Further implementation science research is essential to understand how these interventions can be adapted and scaled effectively across diverse settings, clinical characteristics and populations. This stark discrepancy in attention and action compared to diseases like HIV underscores a critical gap in our public health strategy for DR-TB.

## Lessons from HIV: an integrated approach to DR-TB

The HIV epidemic has illustrated the critical importance of addressing SDoH as part of a comprehensive research and intervention approach. For instance, research indicates that people with cumulative exposure to social and economic disadvantages experience poor engagement in HIV care.^20^ Findings such as these have led to the development of social and structural interventions to improve care engagement critical to ending the HIV epidemic, including tailored adherence counseling, social support interventions, technology-based interventions, comprehensive education programs, expanded healthcare coverage, and economic interventions, such as providing stable housing or cash transfers.^7,8^ Similarly, research has shown that untreated mental health and substance use disorders contribute to poor ART adherence and engagement with HIV prevention and care, spurring efforts to integrate mental health interventions into HIV services.^21^ It is evident that those who experience such challenges also struggle to adhere to TB treatment.^18^ To enhance our response to DR-TB, we must liberate ourselves from a rigid biomedical framework that neglects these significant influences. Just as HIV research has increasingly prioritized individual, social and structural factors that affect health outcomes, TB research must recognize and address these determinants to help foster an environment where individuals are able to adhere to their TB treatment regimens.^22^

## The need for an ecosocial framework

Frameworks developed for HIV care engagement provide invaluable insights that may be translatable to TB care. For example, a heuristic framework has been proposed for HIV acquisition that incorporates individual, social and structural level influences.^15^ This model conceptualizes the social and structural determinants of HIV acquisition and identifies collective risk factors that affect entire communities rather than focusing on individual risk. Since this framework was developed, research has examined these multi-level influences across the HIV care cascade from HIV diagnosis to achieving viral suppression.^15^ Transferring such strategies to the realm of DR-TB could be transformative. The foundation of a successful public health response to DR-TB lies in an ecosocial framework that integrates biological, social and environmental perspectives, emphasizing their connectedness and how they influence each other (see Figure). Ecosocial approaches prompt the examination of the dynamic interplay between social conditions and biological processes, elucidating how they together shape health outcomes.^16^ This integrated perspective is crucial, particularly when considering that treatment interruption is not a binary state. Instead, it is a spectrum of engagement that necessitates a nuanced understanding of patient experiences and the various supports required at different points in the care continuum.

**Figure.**
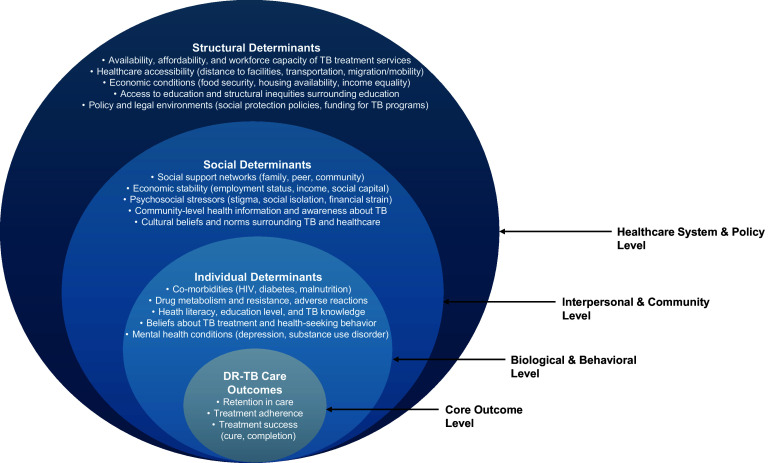
A framework for the social epidemiology of DR-TB care. This framework illustrates the multilevel factors influencing drug-resistant TB (DR-TB) care outcomes across four interrelated levels: the core outcome level, the biological and behavioral level, the interpersonal and community level, and the healthcare system and policy level. The framework highlights the complex interplay among these levels, emphasizing the need for comprehensive, multilevel interventions to improve DR-TB care outcomes.

## CALL TO ACTION

As we confront the complex challenges posed by DR-TB, a commitment to a multifactorial response is paramount, as outlined and agreed upon by Member States at the first UN High-Level Meeting on TB in 2018. We need to implement high-quality, person-centered care. This paradigm shift requires us to build upon lessons learned thus far in improving DR-TB care, alongside a commitment to learning from the successes and setbacks seen in HIV treatment. It is imperative that we close the gaps in TB treatment completion by advancing our understanding of the social and structural determinants that affect retention in care. By reorienting our focus and reimagining our strategies, we can move decisively toward a future where the burden of DR-TB is significantly lessened and ultimately eradicated.
